# Supplementary material for: Stimulus vignetting and orientation selectivity in human visual cortex
Source: eLife. 2018 Aug 14;7:e37241. doi: 10.7554/eLife.37241 (PMC6092116; doi:10.7554/eLife.37241)
Supplement: Transparent reporting form [file elife-37241-transrepform.pdf]

eLIFE
1st Floor
24 Hills Road
Cambridge CB2 1JP, UK
P 01223 855340
W elifesciences.org
T @elife

eLife's transparent reporting form

We encourage authors to provide detailed information *within their submission* to facilitate the interpretation and replication of experiments. Authors can upload supporting documentation to indicate the use of appropriate reporting guidelines for health-related research (see EQUATOR Network), life science research (see the BioSharing Information Resource), or the ARRIVE guidelines for reporting work involving animal research. Where applicable, authors should refer to any relevant reporting standards documents in this form.

If you have any questions, please consult our Journal Policies and/or contact us: editorial@elifesciences.org.

Sample-size estimation

- You should state whether an appropriate sample size was computed when the study was being designed
- You should state the statistical method of sample size computation and any required assumptions
- If no explicit power analysis was used, you should describe how you decided what sample (replicate) size (number) to use

Please outline where this information can be found within the submission (e.g., sections or figure legends), or explain why this information doesn't apply to your submission:

The sample size for this manuscript (N=13) was determined based on prior fMRI studies conducting orientation decoding (e.g., Kamitani & Tong, 2005, Nature Neuroscience (N=4); Freeman et al., 2011, Journal of Neuroscience (N=4); Alink et al., Frontiers in Psychology (N=14); Swisher et al., 2010 (N=4)).

Replicates

- You should report how often each experiment was performed
- You should include a definition of biological versus technical replication
- The data obtained should be provided and sufficient information should be provided to indicate the number of independent biological and/or technical replicates
- If you encountered any outliers, you should describe how these were handled
- Criteria for exclusion/inclusion of data should be clearly stated
- High-throughput sequence data should be uploaded before submission, with a private link for reviewers provided (these are available from both GEO and ArrayExpress)

Please outline where this information can be found within the submission (e.g., sections or figure legends), or explain why this information doesn't apply to your submission:

eLife Sciences Publications, Ltd is a limited liability non-profit non-stock corporation incorporated in the State of Delaware, USA, with company number 5030732, and is registered in the UK with company number FC030576 and branch number BR015634 at the address 1st Floor, 24 Hills Road, Cambridge CB2 1JP | August 2014

1st Floor
24 Hills Road
Cambridge CB2 1JP, UK

P 01223 855340
W elifesciences.org
T @elife

Our study contains both biological (same experiment run on different subjects) and technical (different experiment run on same subjects) replicates, as well as a biological & technical replicate (different experiment run on different subjects). The sinusoidal-modulator experiment was conducted at two facilities (NIH and NYU), on different MRI scanners (3T and 7T), using different scanning parameters and performed on different subject groups. At NIH, all subjects participated in both the sinusoidal-modulator and the square-wave-modulator experiments. This is described under Methods > fMRI experiments. Criteria for inclusion were that subjects performed the task correctly, and the data was not corrupted by dominant artifacts. A single session was discarded due to severe ghosting artifacts, as reported under Methods > fMRI experiments > Observers.

eLife Sciences Publications, Ltd is a limited liability non-profit non-stock corporation incorporated in the State of Delaware, USA, with company number 5030732, and is registered in the UK with company number FC030576 and branch number BR015634 at the address 1st Floor, 24 Hills Road, Cambridge CB2 1JP | August 2014

Statistical reporting

- Statistical analysis methods should be described and justified
- Raw data should be presented in figures whenever informative to do so (typically when N per group is less than 10)
- For each experiment, you should identify the statistical tests used, exact values of N, definitions of center, methods of multiple test correction, and dispersion and precision measures (e.g., mean, median, SD, SEM, confidence intervals; and, for the major substantive results, a measure of effect size (e.g., Pearson's r, Cohen's d)
- Report exact p-values wherever possible alongside the summary statistics and 95% confidence intervals. These should be reported for all key questions and not only when the p-value is less than 0.05.

Please outline where this information can be found within the submission (e.g., sections or figure legends), or explain why this information doesn't apply to your submission:

Maps of orientation selectivity overlaid on flattened cortical surfaces (Fig. 4) were arbitrarily thresholded at a correlation coefficient value of r>0.3. For each voxel the number of samples equals the number of frames in a single scan, i.e. 160. For the circular correlation analysis (Fig. 6), circular correlation coefficients were calculated with custom Matlab scripts. The number of samples for this analysis was the number of V1 voxels pooled across subjects. Statistical significance was computed by permutation testing. The decoding analysis (Fig. 7A) was performed using Matlab function 'classify'. Statistical significance was determined by permutation testing, as described under Methods > fMRI experiments > fMRI MVPA analysis.

(For large datasets, or papers with a very large number of statistical tests, you may upload a single table file with tests, Ns, etc., with reference to sections in the manuscript.)

Group allocation

- Indicate how samples were allocated into experimental groups (in the case of clinical studies, please specify allocation to treatment method); if randomization was used, please also state if restricted randomization was applied
- Indicate if masking was used during group allocation, data collection and/or data analysis

Please outline where this information can be found within the submission (e.g., sections or figure legends), or explain why this information doesn't apply to your submission:

Subjects were not allocated to groups.

Additional data files ("source data")

- We encourage you to upload relevant additional data files, such as numerical data that are represented as a graph in a figure, or as a summary table
- Where provided, these should be in the most useful format, and they can be uploaded as "Source data" files linked to a main figure or table
- Include model definition files including the full list of parameters used
- Include code used for data analysis (e.g., R, MatLab)

- Avoid stating that data files are "available upon request"

Please indicate the figures or tables for which source data files have been provided:

We have not currently provided data for figures. We plan to provide data for all figures upon publication.
